# Influence of genotype, floral stage, and water stress on floral nectar yield and composition of mānuka (*Leptospermum scoparium*)

**DOI:** 10.1093/aob/mcx183

**Published:** 2018-01-02

**Authors:** Michael J Clearwater, Maria Revell, Stevie Noe, Merilyn Manley-Harris

**Affiliations:** School of Science, University of Waikato, Hamilton, New Zealand

**Keywords:** Nectar, mānuka, genotype, environment, *Leptospermum scoparium*, dihydroxyacetone, water stress, honey, composition, floral stage

## Abstract

**Background and Aims:**

Floral nectar can be variable in composition, influencing pollinator behaviour and the composition of honey derived from it. The non-peroxide antibacterial activity of mānuka (*Leptospermum scoparium*, Myrtaceae) honey results from the chemical conversion of the triose sugar dihydroxyacetone (DHA), after DHA accumulates for an unknown reason in the nectar. This study examined variation in nectar DHA, glucose, fructose and sucrose content with floral stage of development, between mānuka genotypes with differing flower morphology, and in response to water stress.

**Methods:**

Six mānuka genotypes were grown without nectar-feeding insects. Stages of flower development were defined, nectar was harvested and its composition was compared between stages and genotypes, and with floral morphology. Water stress was imposed and its effect on nectar composition was examined.

**Key Results:**

Nectar was present from soon after flower opening until the end of petal abscission, with the quantity of accumulated nectar sugars rising, then stabilizing or falling, indicating nectar secretion followed by reabsorption in some genotypes. The quantity of DHA, the ratio of DHA to other nectar sugars and the fructose to glucose ratio also varied with stage of development, indicating differences in rates of production and reabsorption between nectar components. Nectar composition and yield per flower also differed between genotypes, although neither was positively related to nectary area or stomatal density. Drying soil had no effect on nectar composition or yield, but variation in nectar yield was correlated with temperature prior to nectar sampling.

**Conclusions:**

Mānuka nectar yield and composition are strongly influenced by plant genotype, flower age and the environment. There were clear stoichiometric relationships between glucose, fructose and sucrose per flower, but DHA per flower was only weakly correlated with the amount of other sugars, suggesting that accumulation of the triose sugar is indirectly coupled to secretion of the larger sugars by the nectary parenchyma.

## INTRODUCTION

Floral nectar composition and volume are often variable between flowers and plants of a given species (Pacini and Nepi, 2007). Composition for a given species is usually dominated by the major sugars glucose, fructose and sucrose in relatively consistent proportions, but a wide variety of other compounds may also be present in variable amounts (Carter and Thornburg, 2004; Nicolson and Thornburg, 2007). Nectar volume and concentration are also often highly variable between flower developmental stages, flowers and individual plants, and in response to biotic and abiotic factors (Mitchell, 2004). The floral nectars of mānuka (*Leptospermum scoparium*) and some other *Leptospermum* species are dominated by fructose and glucose, but also contain small but variable amounts (usually <2 % each) of sucrose and the three-carbon sugar dihydroxyacetone (DHA) (Adams *et al.*, 2009; Norton *et al.*, 2015; Nickless *et al.*, 2016). Nectar DHA confers the sought-after non-peroxide antibacterial activity of mānuka honey, after it converts to methylglyoxal during maturation of the honey (Adams *et al.*, 2008; Mavric *et al.*, 2008). Despite a large industry developing around the unique properties of mānuka honey, little is known about the controls on nectar flow and composition in this species. Surveys of nectar composition from both wild and cultivated mānuka have revealed significant variation in both total nectar sugar amount per flower, and the ratio of DHA to the major sugars (Williams *et al.*, 2014; Nickless *et al.*, 2017), but the cause of this variation and the origin of the nectar DHA both remain unknown.

**Fig. 1.**
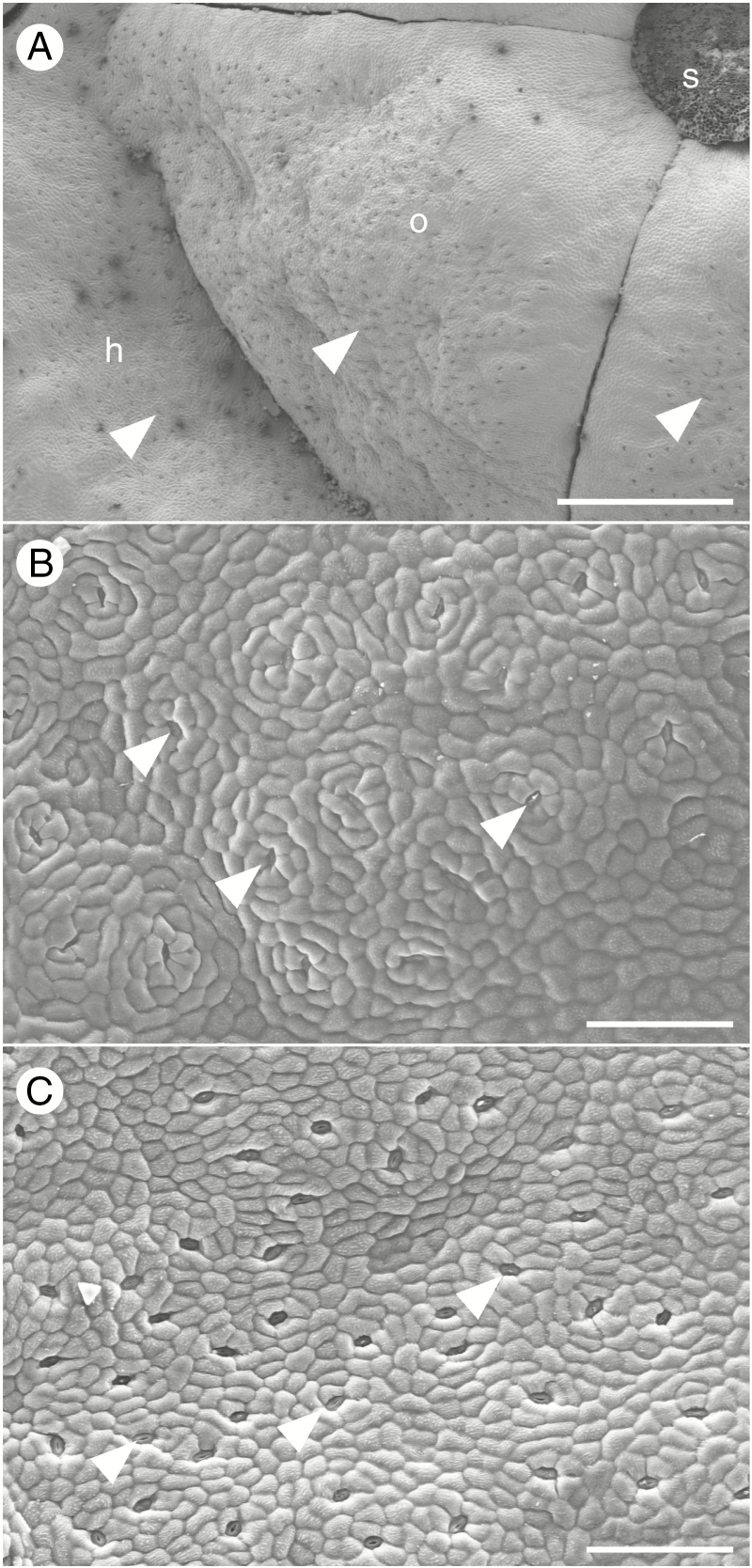
SEM micrographs of the nectary surface of nectar-secreting mānuka flowers at Stage 3. (A) Base of the excised style (s), upper surface of the ovary (o) and inner wall of the hypanthium (h) of an NT flower. Modified stomata (arrowheads) are visible on the ovary and hypanthium surface. (B,C) Examples of variation in the density of modified stomata (arrowheads) between genotypes (B, MI; C, WK) Scale bars = 500 µm in A, 100 µm in B and C.

Mānuka is a fast growing shrub indigenous to New Zealand, usually found as a short-lived colonist of disturbed habitats, or dominating poorly drained and extremely infertile habitats (Stephens *et al.*, 2005). The species is andromonoecious, producing long-lived (7–21 d) perfect and male flowers in variable proportions during spring and summer (Primack and Lloyd, 1980; Primack, 1980). The morphology of the floral nectary is similar to that of other members of the Myrtaceae, with nectar readily observed to accumulate on the inner surface of the hypanthium and on the upper surface of the gynoecium (O’Brien *et al.*, 1996; Davis, 1997). This zone corresponds with the location of modified stomata (Fig. 1A), through which nectar exudes in other myrtaceous species, from intercellular spaces amongst the nectary parenchyma (Davis, 1997). Nectar is produced throughout the life of the flower in other *Leptospermum* species (O’Brien and Calder, 1993), from soon after opening until petal fall. When pollinators were excluded from flowers of three *Eucalyptus* species, standing nectar volumes peaked as the flowers aged, then declined, providing evidence for nectar reabsorption in the Myrtaceae (Davis, 1997); nectar composition (glucose, fructose and sucrose) did not change as the flowers aged or when pollinators were excluded, but differed between species. Nectar yield was correlated with flower size but not stomatal density between species (Davis, 1997). Nectar yields per flower, hexose ratios, and sucrose and DHA contents are known to vary between mānuka genotypes, plants and with date of collection (Williams *et al.*, 2014; Nickless *et al.*, 2017). Some of this variation can probably be attributed to flower age, pollinator activity or environmental conditions, but the effects of these variables have not been investigated in mānuka.

**Fig. 2.**
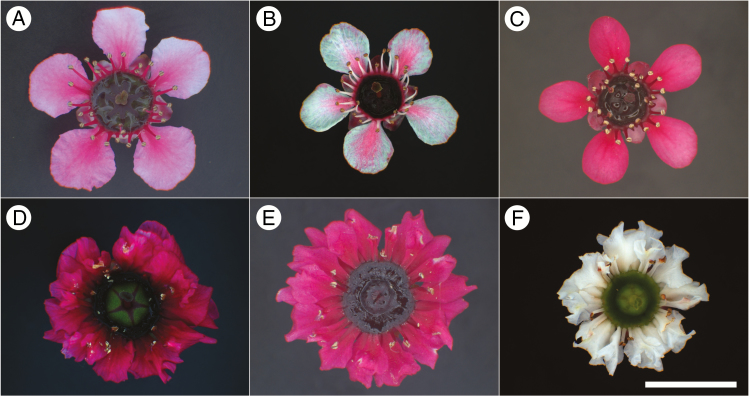
Morphology of Stage 3 open flowers of the six mānuka (*Leptospermum scoparium*) genotypes used in this study. (A) ‘Martinii’ (MI); (B) ‘Nanum Tui’ (NT); (C) ‘Red Ensign’ (RE); (D) ‘Red Damask’ (RD); (E) ‘Wiri Kerry’ (WK); (F) ‘Snow Flurry’ (SF). Scale bar = 10 mm.

The goal of this study was to define floral developmental stages for the mānuka flower and compare changes in nectar yield and composition as the flowers aged, between genotypes that varied strongly in flower morphology, and in response to water stress. Floral stages have been defined previously for the study of nectar production by *Eucalyptus* flowers (Davis, 1997; Nickless *et al.*, 2017), but these are not directly applicable to mānuka flowers because the *Eucalyptus* flower produces an operculum rather than petals, and gradually unfurls multiple whorls of stamens, rather than the more discrete opening of the corolla and androecium that occurs in mānuka. Six readily available *L. scoparium* ornamental genotypes (cultivars) were selected that differed in flower size, corolla colour (white to pink/red) and whether they carried a double-petal mutation (Dawson, 2010*a*; Fig. 2). Mānuka is a highly variable species and has long been a target of selection and some deliberate crossing for desirable ornamental characteristics, resulting in many named cultivars with high flower numbers and extended flowering periods, as well as a variety of growth forms (Stephens *et al.*, 2005; Dawson, 2010*b*). With the current high value of mānuka honey there is now increasing interest in the selection of genotypes with high nectar sugar production and high levels of nectar DHA (Nickless *et al.*, 2014, 2017). However, relatively few woody angiosperms have been investigated for genotypic variation in nectar traits or in flower structure that might relate to nectar traits (Shuel, 1989; Davis, 2001).

The plants were grown under well-watered conditions in a common glasshouse and nectar was harvested from the developmental stages that clearly produced nectar. We then investigated whether nectar yield or composition changed when the plants were subjected to soil moisture stress. It was hypothesized that nectar composition, including hexose ratios and the ratio of DHA to the other sugars, would differ between genotypes but vary little with flower age, whilst the standing nectar yield per flower was expected to peak then decline as the flower aged. Drought can reduce nectar volume or concentration in other species (Nicolson and Thornburg, 2007). It was therefore expected that there would be a decline in nectar yield (measured as sugar content per flower) and an increase in the ratio of DHA to sugar with soil drying, based on the idea that changes in carbohydrate metabolism associated with stress could cause increased accumulation of a low-molecular-weight solute such as DHA (Hare *et al.*, 1998).

## MATERIAL AND METHODS

### Plant material and growing conditions

Ten clonally replicated plants of each of six named genotypes of *Leptospermum scoparium* J.R.Forst. et G.Forst. were purchased from a nursery (Annton Nursery Ltd, Tamahere, New Zealand) and repotted into 5-litre pots using a standard commercial potting mix (GB Mix, Daltons Ltd, Hinuera, New Zealand; 65 % bark fibre and fines, 15 % coco fibre, 20 % pumice, slow-release fertilizers and trace elements). The genotypes were selected based on their contrasting flower morphologies, mixed parentage and prior knowledge of likely nectar DHA content (Williams *et al.*, 2014). *Leptospermum scoparium* ‘Nanum Tui’ (NT) and ‘Red Ensign’ (RE) are diploid genotypes with a single whorl of white/pink and red petals, respectively (Fig. 2). ‘Martinii’ (MI) is a triploid genotype with a large flower and a single whorl of white petals that turn pink after the flower opens (Dawson, 2010*b*; Fig. 2). In New Zealand, ‘Martinii’ is readily available from commercial nurseries, but is usually mislabelled as its maternal parent, the tetraploid genotype ‘Keatleyii’ (Dawson, 2010*a*). The remaining three diploid genotypes (‘Red Damask’, red petals; ‘Wiri Kerry’, red petals; ‘Snow Flurry’, white petals; abbreviated RD, WK and SF, respectively) all carry a ‘double flower’ mutation that results in multiple whorls of petals, and a reduced number of stamens (Fig. 2). The parentage of these cultivars is uncertain, partly because open pollinated seed has often been used within ornamental *Leptospermum* breeding programmes. However, the red to pink petal coloration of RE and MI is believed to result from a shared parent, the red-flowered and wild selected ‘Nichollsii’ (Dawson, 2010*b*). Similarly, the red petals of WK and RD may also derive ultimately from an earlier cross with ‘Nichollsii’, whilst the double flower mutation shared by WK, SF and RD may originate, directly or indirectly, from the same double-flowered parent cultivar ‘Flore Pleno’ (Dawson, 2010*b*). NT is derived from an open-pollinated, possibly wild-selected seed parent ‘Nanum’ (Dawson, 2010*a*, b), and may therefore be the cultivar that is the least related to the other five used in this study. The plants were grown in an automated glasshouse during the flowering period from September to November 2013, with natural lighting (11- to 14-h day length), and ventilation and automated shade screens set to start when air temperature reached 20 °C. Pollinators and all other nectar-feeding insects, including ants, were excluded throughout. During this period room temperature varied naturally between 8 and 22 °C depending on external conditions, with temperature, relative humidity and external radiation recorded continuously by the glasshouse control system (Synopta, Hortimax, the Netherlands). All plants were watered daily to field capacity, except during the drought experiment.

### Flower phenology and morphology

**Fig. 3.**
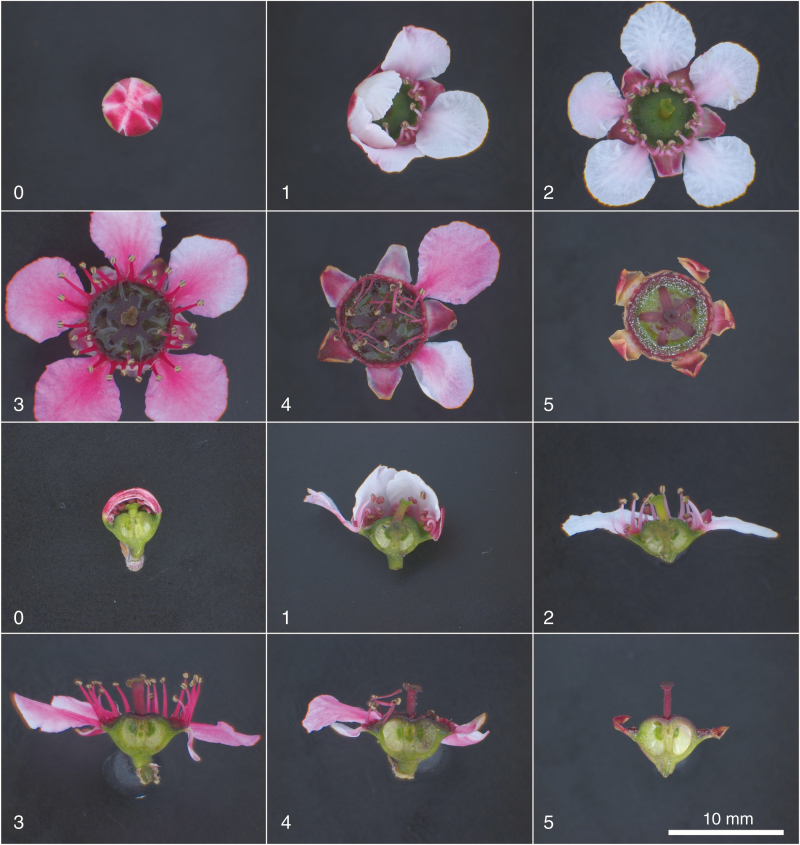
Stages of mānuka flower development in plan (above) and half flower view (below), for the MI genotype, from flower opening until sepal fall. Numbers correspond to the floral stages defined in Box 1.

During September and early October, flower buds were randomly selected from all plants, tagged and observed as they progressed from opening to petal and sepal fall. Six stages (see Box 1) of flower development were defined based on flower morphology, imaged using a dissecting microscope and camera, and shown in Fig. 3 for genotype MI.

**Box 1. Six stages of flower development**

**Stage 0** (Flower Bud): A recognizable flower bud, with sepals and petals visible but unopened.
**Stage 1** (Petal Opening): Begins when first petal starts to reflex. The ovary and hypanthium remain at least partially obscured and stamens folded.
**Stage 2** (Stamen Unfurling): Begins with all petals reaching erect to horizontal position, but at least some stamens remain partially furled. During this stage the style is lengthening but the stigma remains below the height of the tallest stamens. Nectar secretion and anther dehiscence begin. The ovary and hypanthium begin to darken from green to red in some genotypes.
**Stage 3** (Open Flower): Begins when all stamens are no longer folded, and have reached vertical or spreading position. Petals fully reflexed; stigma at a similar height or higher than anthers; nectar secretion continues; hypanthium red in some genotypes.
**Stage 4** (Petal Drop): Begins when first petal abscises. Stamens begin to shrivel, and nectar may still be present.
**Stage 5** (Sepal Drop): Begins when the first sepal abscises. All petals have abscised, and nectar residues may or may not be present.

Stage 3 in this description corresponds approximately to stage 4 in the description of Davis (1997), which is based on prolonged unfurling of *Eucalyptus* stamens. The number of days required for flowers to progress through stages 1–4 was recorded for 30 tagged flowers. Ten nectar samples were collected from untagged flowers with morphologies corresponding to floral stages 2–4, on a range of dates, with each sample containing nectar washed from five flowers from the same plant. The petals of each flower were marked at sampling, and each flower was sampled only once during development, so that the harvested nectar represented the cumulative standing nectar amount for that developmental stage in the absence of nectar-feeding insects.

Genotype averages for nectary surface area and stomatal density were compared with nectar yield and composition by linear regression. A thick median longitudinal section was cut from each of five flowers of Stage 3 per genotype and imaged with a dissecting microscope. Image analysis (Schneider *et al.*, 2012) was used to measure the lengths and heights of the upper surface of the ovary and inner wall of the staminophore, and nectary area was estimated as the sum of the surface area of three conical frustrums representing the staminophore and ovary lateral and upper surfaces. A further six dissected flowers of Stage 3 were fixed under vacuum in formalin, acetic acid and alcohol fixative, dehydrated through a graded alcohol series, dried using a critical point drier and mounted nectary surfaces upwards on stubs using graphite tape. Samples were sputter coated with platinum (Hitachi E-1030 Ion Sputter Coater) and imaged with a scanning electron microscope (Hitachi S-4700) at 20 keV. Nectary stomatal density was estimated separately for the staminophore wall and ovary surface, from counts of stomata visible within one to five random fields of view per flower.

### Effect of water stress

After flower phenology observations were completed, five plants of each genotype were randomly assigned to each of well-watered and water-stressed treatments. The irrigation rate of the five water stress plants was gradually reduced over a 7-d period, starting on 12 October, to achieve a predawn shoot water potential of −0.3 MPa, measured using a pressure chamber (1505D-EXP, PMS Instrument Company, Albany, OR, USA). Preliminary experiments with identical plants were required to identify the minimum water potential and associated pot weights that could be tolerated without causing shoot loss. Mortality occurred within 2 d if watering was withheld completely and water potentials were allowed to fall to more negative values. Water-stressed plants were hand watered daily to their target weight, and held at their target weight for a further 10 d, before re-watering to field capacity. Well-watered plants were watered daily to field capacity. Genotypes and treatments were arranged in a randomized complete block design, with five blocks (glasshouse tables). One pooled sample of accumulated nectar was harvested from five flowers at Stage 3 per plant, with half of all the plants sampled each day, so that all plants were sampled every second day. Reported values are the average of the five plants for each treatment × genotype combination, for each 2-d period. Sampled flowers were marked and excluded from further sampling.

### Nectar harvesting and chemistry

Nectar was harvested non-destructively between dawn and midday by pipetting 200 µL (four 50-µL aliquots) of deionized water into the floral cup formed by the hypanthium and ovary, and recovering these with a pasteur pipette, with samples pooled for a total of five flowers per plant. Samples were immediately placed on ice then transferred as soon as possible to storage at −20 °C until analysis. Nectar wash samples were derivatized and analysed by GC-FID (gas chromatography with flame ionization detector) as previously reported (Williams *et al.*, 2014). Briefly, nectar wash (200 µL) was derivatized with *O*-(2,3,4,5,6-pentafluorobenzyl) hydroxylamine hydrochloride and subsequently the organic-soluble product of the first derivatization was further derivatized with 1-(trimethylsilyl)imidazole (TMSI) to allow GC-FID analysis of DHA. Nectar wash (20 µL) was derivatized with TMSI for GC-FID analysis of nectar sugars; per-*O*-TMS glucose, fructose and sucrose were identified based on retention times and mass spectral fragmentation.

Analysis of samples from the flower phenology observations quantified fructose, glucose and DHA per flower. Analysis of samples from the water stress experiment also included quantification of sucrose per flower.

### Statistical analysis

All analyses were conducted in R (R Core Team, 2016). The effects of genotype on floral stage duration (phenology experiment), mean nectar variables (water stress experiment) and flower morphology (nectar area and stomatal density measurements) were tested with a one-way ANOVA, with *post-hoc* mean separation using Tukey’s honest significant difference (HSD) test. Linear models with Gaussian error distributions were used to test for genotype and floral stage effects on nectar sugar yields (mg per flower) and ratios (fructose to glucose ratio, DHA/sugar, where sugar was the sum of glucose and fructose) in the phenology experiment. Models were fitted using the lm function, with Type II SS calculated using the ANOVA function in package CAR. Log and square root transformations were used as required to improve normality before analysis. The six genotypes clearly separated into four that produced significant amounts of nectar, and two (SF and RD) that produced little to no nectar. Therefore, models were also fitted without the inclusion of SF and RD, to examine floral stage and genotype effects when significant amounts of nectar were produced. A similar analysis was applied to the results from the water stress experiment, with irrigation treatment, genotype and date modelled as fixed effects on nectar variables. No irrigation treatment was detected, but an up to five-fold variation in nectar sugar per flower with sampling date was observed, prompting a cross correlation analysis with and without time lags between daily nectar variables and glasshouse-recorded environmental variables (temperature, vapour pressure deficit, external radiation), using the ccf function.

## RESULTS

Under glasshouse conditions the six genotypes differed significantly (*P* < 0.001) in the number of days for completion of each floral stage, with the overall time from first petal opening (Stage 1) to complete petal abscission (end of Stage 4) varying from 18 to 27 d (Table 1). In general, flower opening (Stage 1) occurred rapidly, within 1–3 d (slower in the double-petal genotypes SF and RD), and the longest stage was Stage 3, with the flower fully open and nectar secretion continuing (Fig. 3).

**Table 1.** Average length in days of floral stages 1–4, in the absence of pollinators (±s.e.) for six genotypes of mānuka

Genotype	Floral stage			
	Stage 1	Stage 2	Stage 3	Stage 4
Martinii (MI)	3.6 ± 0.1^b^	6.4 ± 0.2^d^	8.3 ± 0.2^b^	9.1 ± 0.1^d^
Nanum Tui (NT)	2.0 ± 0.1^a^	5.6 ± 0.2^bc^	10.4 ± 0.4^c^	6.9 ± 0.3^c^
Red Ensign (RE)	2.5 ± 0.1^a^	3.9 ± 0.2^a^	7.9 ± 0.2^b^	4.5 ± 0.2^b^
Red Damask (RD)	4.3 ± 0.1^c^	6.0 ± 0.2^cd^	6.5 ± 0.2^a^	3.4 ± 0.1^a^
Snow Flurry (SF)	5.5 ± 0.2^d^	6.6 ± 0.3^d^	6.9 ± 0.1^a^	3.3 ± 0.1^a^
Wiri Kerry (WK)	2.5 ± 0.1^a^	5.1 ± 0.2^b^	10.2 ± 0.3^c^	7.3 ± 0.2^c^

Stage length varied between genotypes for all four stages (ANOVA, *P* < 0.001). Values within each stage that share a letter were not significantly different (Tukey’s HSD, *P* > 0.05).

**Fig. 4.**
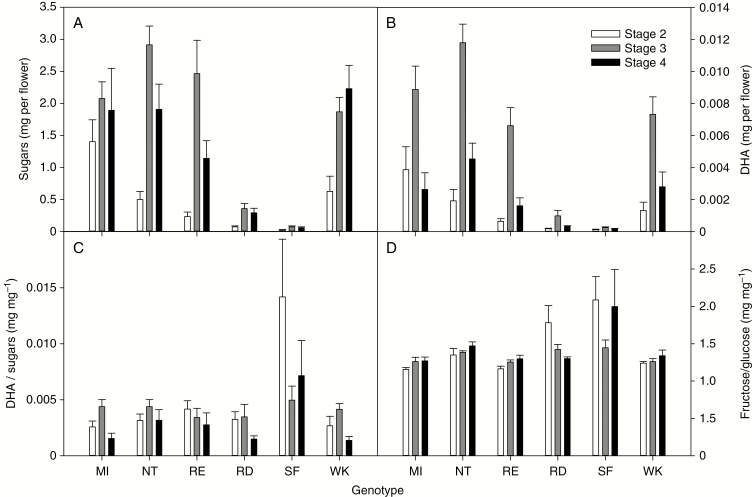
Nectar yield (total sugars per flower and DHA per flower) and composition (DHA to sugars and fructose to glucose ratios) for six genotypes of mānuka for floral stages 2–4. For genotype abbreviations refer to the legend for Fig. 2. Values are means ± s.e.

The amount and composition of nectar varied between genotypes and floral stages. The amount of accumulated nectar sugar was highest at Stage 3 (*P* < 0.001), before declining in at least two of the six genotypes (NT and RE) during Stage 4 (Fig. 4A), suggesting that nectar reabsorption can occur if pollinators are not present. This temporal pattern was matched by the accumulation of nectar DHA (*P* < 0.001), except that DHA levels fell more sharply than sugars in all genotypes at Stage 4, suggesting faster reabsorption or degradation of DHA compared to glucose and fructose. Temporal differences in production and reabsorption rates between hexoses and DHA contributed to a more pronounced peak in DHA/sugar ratio during Stage 3 (*P* < 0.01). Two of the double-petal genotypes (SF and RD) produced significantly less nectar overall (Fig. 4A), but a similar pattern of peak accumulated nectar during Stage 3 (*P* < 0.001). Nectar production was lowest and the DHA/sugar ratio highly variable in SF. Compared to nectar amounts, fructose/glucose ratios were relatively stable, but differed between genotypes (*P* < 0.001) (Fig. 4D).

When the two low nectar-producing genotypes were excluded from the analysis, differences in the amount of nectar sugar per flower were still present (*P* = 0.01), but there were no differences in DHA per flower (*P* = 0.06) or the ratio of DHA/sugar (*P* = 0.3) between the four remaining genotypes (Fig. 4A–C). However, the influence of floral stage remained highly significant for all of these variables (*P* < 0.001). With all genotypes included in the analysis, the ratio of fructose to glucose in the nectar differed between genotypes (*P* < 0.001), and increased from Stage 2 to 4 (*P* < 0.01), particularly in high nectar-producing genotypes (Fig. 4D). There were no interactions between floral stage and genotype in their effect on these variables.

**Fig. 5.**
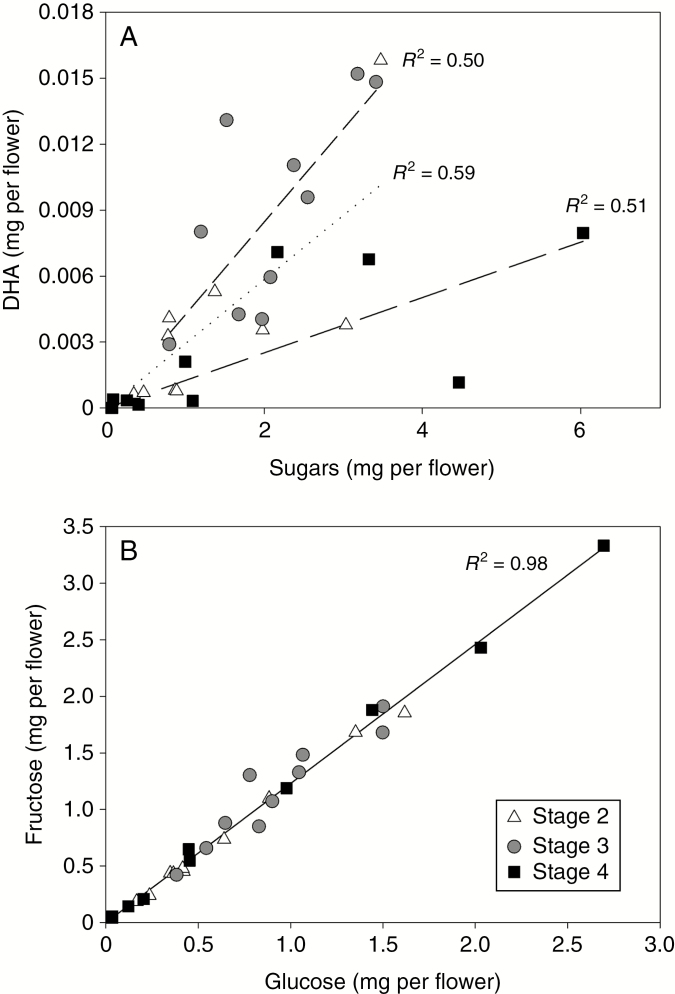
Relationships between (A) the amount of DHA per flower and fructose plus glucose per flower and (B) quantities of fructose and glucose per flower, for nectar harvested from mānuka genotype MI for floral stages 2–4.

When individual nectar samples were considered, the amount of DHA present was clearly correlated with the presence of the two hexose sugars, but the ratio of DHA to sugars was variable from sample to sample within a genotype, in addition to variation with stage of development (Fig. 5A, shown for MI). In comparison, the amounts of fructose and glucose present at any time were much more closely correlated with each other, in proportions that differed little between floral stage (Fig. 5B), except when nectar yields were low (Fig. 4D). Similar patterns were observed for all six genotypes (data not shown).

**Fig. 6.**
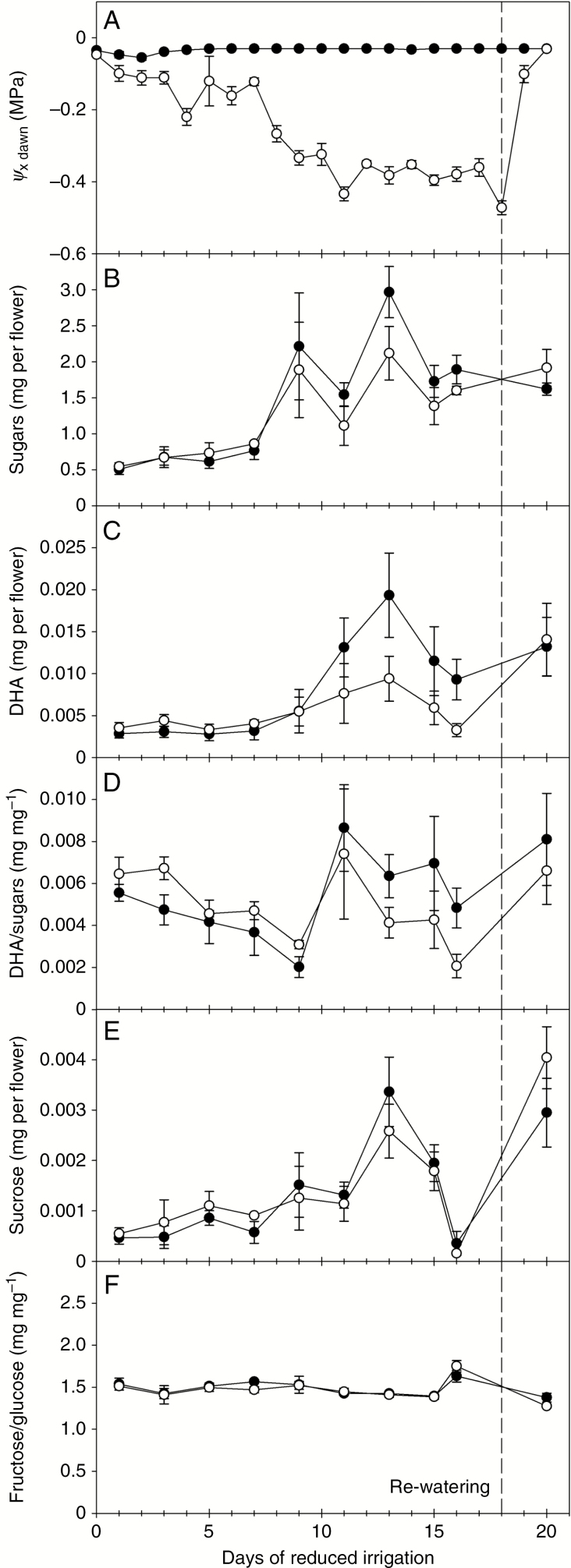
Effect of drying soil on predawn xylem water potential (Ψ_x dawn_) and nectar composition for flowers of mānuka genotype MI at Stage 3. Irrigation was reduced in stressed plants (open symbols) from day 0, while non-stressed plants (closed symbols) were irrigated daily to soil field capacity. Stressed plants were returned to full irrigation on day 18. Values are means ± s.e. Results for the other five genotypes are provided in Supplementary Data Figs S1–S5.

Across all genotypes, drying soil had no significant effect on nectar yield or composition from Stage 3 flowers (*P* > 0.05 for all variables), although both varied significantly with day of collection (*P* < 0.01) and between genotypes (*P* < 0.001) (Fig. 6 for genotype MI; Supplementary Data Figs S1–S5 for the other five genotypes). Average nectar yields per Stage 3 flower (Table 2) during this experiment were approximately half or less compared to that from the earlier phenology measurements (Fig. 4A), using the same cohort of plants, and fructose to glucose ratios were higher (Fig. 4D). Nectar sugars and DHA per flower increased after day 7 of the experiment in both well-watered and drought-affected plants of all genotypes that produced sufficient nectar for measurement (Figs 6 and S1–S5). The DHA/sugar ratio varied with date (*P* < 0.001) but was unaffected by drought (*P* = 0.53). The average ratio for both treatments differed significantly between genotypes (*P* < 0.001; Table 2), more so than in the earlier experiment (Fig. 4C), whilst the ratio of fructose to glucose remained stable at a value that differed between genotypes (*P* < 0.001). Nectary area, and the density of modified stomata on the hypanthium and ovary surface differed between genotypes (*P* < 0.001 for all variables; Fig. 1 and Table 2). Across genotypes there was always a higher stomatal density on the hypanthium than on the ovary upper surface (*P* < 0.01, Table 2). Nectar yield per flower decreased with increasing nectary surface area (linear regression, *R*^2^ = 0.79, *P* = 0.02, data not shown), but this relationship was driven primarily by the two low nectar-producing genotypes (RD and SF), which had the broadest ovary and hypanthium surface areas (Table 2). Apart from this effect, there were no clear relationships between nectary surface areas or stomatal density, and nectar yield per flower or nectar composition (*P* > 0.05).

**Table 2.** Nectary surface area (*A*_nectary_), density of modified stomata (*D*) of the ovary and hypanthium surfaces, and mean nectar properties during the water stress experiment, for six genotypes of mānuka

Variable	Genotype					
	MI	NT	RE	RD	SF	WK
*A*_nectary_ (mm^2^)	100 ± 5^abc^	73 ± 6^a^	82 ± 6^ab^	115 ± 8^bc^	134 ± 15^c^	117 ± 9^bc^
*D*_ovary_ (mm^−2^)	114 ± 3^a^	262 ± 6^c^	162 ± 8^ab^	156 ± 24^ab^	125 ± 7^ab^	174 ± 10^b^
*D*_hypathium_ (mm^−2^)	123 ± 8^a^	280 ± 15^d^	199 ± 19^c^	180 ± 16^bc^	142 ± 7^ab^	214 ± 9^c^
Sugars (mg per flower)	1.36 ± 0.09^e^	0.85 ± 0.06^d^	0.54 ± 0.06^c^	0.14 ± 0.03^b^	0.01 ± 0.00^a^	0.69 ± 0.05^cd^
DHA/sugar (mg mg^−1^)	0.0054 ± 0.0003^b^	0.0035 ± 0.0003^ab^	0.0040 ± 0.0003^ab^	0.0016 ± 0.0004^a^	0.0019 ± 0.0011^a^	0.0035 ± 0.0004^ab^
Sucrose (µg per flower)	1.50 ± 0.14^d^	0.84 ± 0.11^bc^	1.32 ± 0.19^cd^	0.49 ± 0.13^b^	0.14 ± 0.06^a^	43.8 ± 0.41^e^
Fructose/glucose (mg mg^−1^)	1.46 ± 0.01^a^	1.55 ± 0.01^ab^	1.49 ± 0.02^ab^	1.67 ± 0.06^ab^	2.49 ± 0.55^b^	1.49 ± 0.01^ab^

During the water stress experiment there were significant differences between genotypes in nectar sugar per flower, DHA/nectar sugar, nectar sucrose per flower and fructose to glucose ratio in nectar during the experiment (*P* < 0.05). Means (±s.e.) in the same row followed by different letters were significantly different (*P* < 0.05, Tukey’s HSD). For genotype abbreviations refer to the legend for Table 1.

**Fig. 7.**
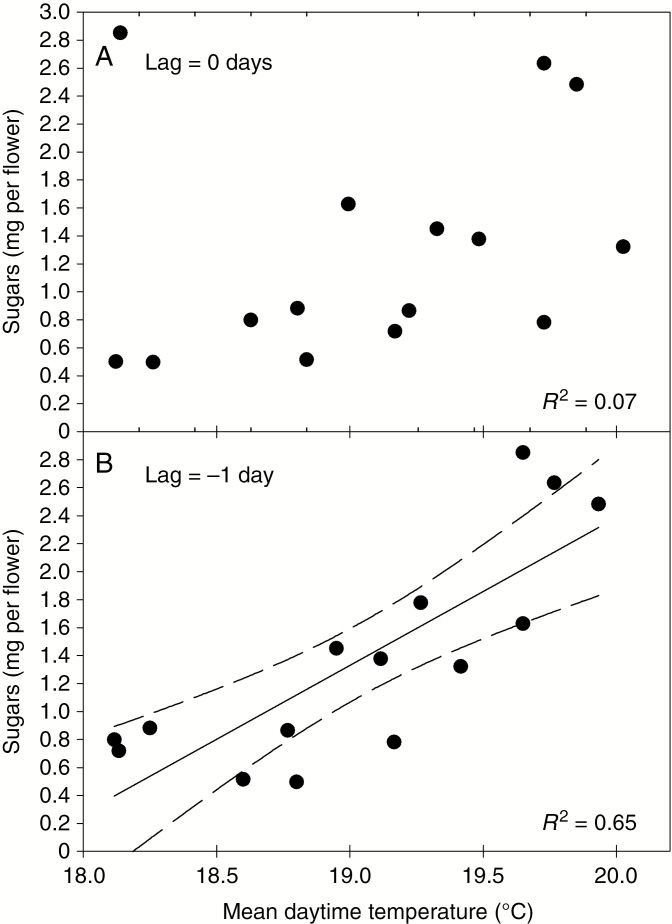
Relationship between average nectar yield per flower from Stage 3 flowers of mānuka genotype MI during the water stress experiment (both irrigation treatments), and daytime air temperature on the day of nectar collection (A), or daytime air temperature on the day before nectar collection (B).

Cross correlation analysis revealed that at least some of the variation in nectar flow (sugars per flower) during the water stress experiment appeared to be related to temperature, with nectar sugars positively correlated with daytime temperature of the day before rather than the day of collection (*P* < 0.001; Fig. 7, shown for MI; the correlation was also significant with all four high nectar-producing genotypes included). Some of the increase in nectar flows during the latter half of the experiment can be attributed therefore to small increases in daytime temperature during this period. Temporal variation in nectar composition (DHA/sugar and fructose to glucose ratios) was not related to any environmental variable, and no other environmental variable was significantly correlated with nectar amount.

**Fig. 8.**
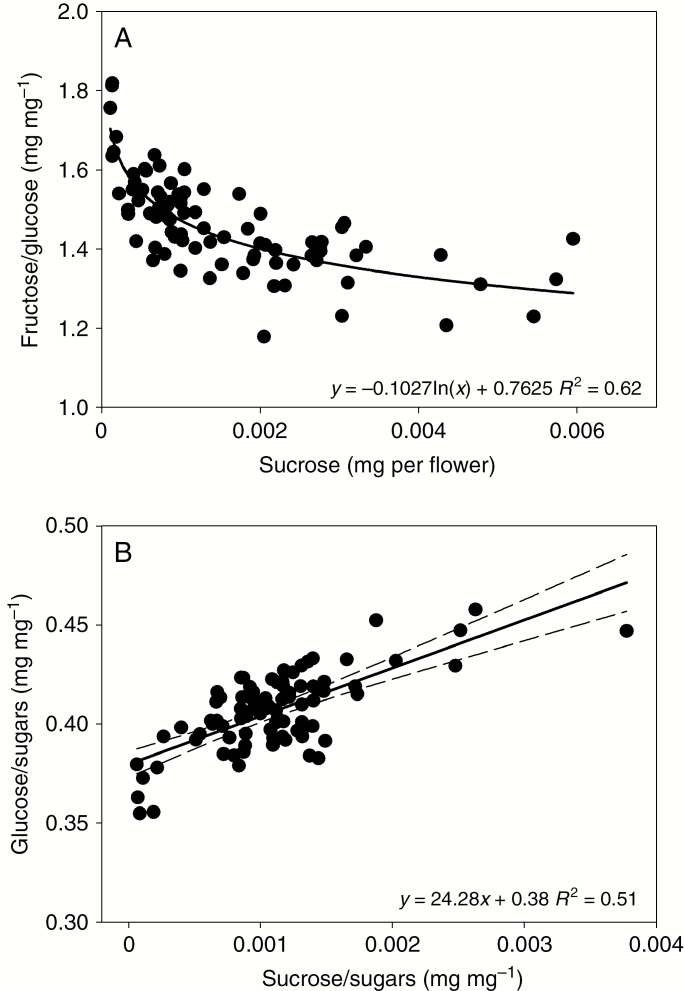
Relationships between glucose, fructose and sucrose content of nectar from Stage 3 flowers of mānuka genotype MI during the water stress experiment. (A) Fructose to glucose ratio as a function of sucrose content for the same samples. (B) Glucose to total sugar (glucose, fructose and sucrose) ratio as a function of the sucrose to total sugar ratio. Each point represents the pooled nectar from five flowers from the same plant.

Sucrose was detected in low concentrations in nectar samples from the drought experiment (usually around 0.1 % of total sugars; Table 2). The genotypes differed significantly in sucrose per flower and the relative contribution of sucrose to total sugars, with much higher sucrose content in nectar from WK (*P* < 0.001; 6 % of total sugars; Table 2). No relationship was detected between nectar DHA and sucrose contents across individual nectar samples, either within or between genotypes. However, sucrose content was associated with changes in fructose to glucose ratio. Between samples from a given genotype, as sucrose content increased, the fructose to glucose ratio decreased (*P* < 0.001; Fig. 8A), resulting in a linear relationship between the relative contributions of glucose and sucrose to total nectar sugar content (*P* < 0.001; Fig. 8B, shown for MI; similar patterns were observed for all four high-producing nectar genotypes).

## DISCUSSION

Nectar yield and composition from the mānuka flower were clearly influenced by plant genotype, the stage of flower development and environmental conditions, particularly temperature. Nectar yield increased and declined with stage of development, as hypothesized, but there were also unexpected and predictable changes in nectar composition as the flower progressed from opening to the beginning of capsule formation. The hypothesis that drought would reduce nectar yield and alter nectar composition was not supported. The genotypes could be broadly separated into those with low and high nectar yields per flower, and differed consistently in nectar composition, but yields and DHA content relative to the other nectar sugars were also highly variable within each genotype. The results provide new insight into the mechanism of nectar secretion by the mānuka floral nectary, suggesting the secretion of sucrose and its near-complete hydrolysis to hexoses. In contrast, it is hypothesized that DHA has a different origin to the larger sugars, as a by-product of primary metabolism within the nectary parenchyma.

Substantial genetic control over intraspecific variation in floral nectar traits has been described previously for a limited number of species, but often only for variation in nectar volume. A small number of studies have quantified strong heritable variation in nectar production (Mitchell, 2004), and clonal variation in nectar yield and chemistry has been documented in crop plants, particularly *Brassica napus* L. var. *oleifera* (Kevan *et al.*, 1991; Pierre *et al.*, 1999; Bertazzini and Forlani, 2016). Surprisingly, even though clones of only six genotypes were compared, total nectar sugar per flower for a given floral stage varied by two orders of magnitude between the lowest and highest producers, with consistent separation between four high-yielding genotypes, and two that produced only small amounts of nectar. The range of nectar yields and composition overall were comparable to those reported previously for mānuka (Williams *et al.*, 2014; Nickless *et al.*, 2016, 2017). Amongst genotypes, SF and RD may share a low nectar yielding trait because they are likely to be siblings from the *F*_2_ progeny of a cross between a red single-petal seed parent and a pink double-petal pollen parent; they also share the double-petal mutation of the pollen parent, but differ in coloration (Dawson, 2010*b*). Low nectar flow is not clearly linked to the double-petal character, because the third double-petal genotype (WK) produced higher yields of nectar and arose from a separate selection programme that included similar parentage (Dawson, 2010*b*). However, ornamental double-flower mutants of common herbaceaous ornamentals frequently secrete little or no nectar (Comba *et al.*, 1999; Corbet *et al.*, 2001). Overall the results suggest that there is heritable variation in nectar traits in mānuka, but also that for these genotypes there are no clear associations between the floral morphological traits considered (petal number, colour, hypanthium size, stomatal density) and nectar yield or composition. Genotypic variation in nectar flow may therefore be controlled primarily by the metabolism of the underlying nectary tissue, or anatomical traits such as phloem vascularization or nectary tissue volume (Davis, 2001).

Nectar composition, in terms of relative amounts of hexose, sucrose and DHA, varied less than total sugar amount between genotypes. Most between-genotype variation in DHA/sugar and fructose to glucose ratios was associated with low nectar-yielding genotypes (SF and RD) and floral stages, a possible artefact of collection and analysis of small nectar volumes. Sucrose was a minor constituent of nectar sugars, but sucrose content varied strongly and consistently between genotypes, a trait that could potentially influence honey bee preference (Wykes, 1952). Together these observations of genotypic differences in nectar yield and composition indicate that heritable variation in floral nectar yield and composition does occur in mānuka, as previously inferred from nectar variation observed in natural and cultivated populations of mānuka (Williams *et al.*, 2014; Nickless *et al.*, 2017). However, confirmation of the potential for natural or artificial selection on nectar traits in this species awaits a more formal genetic investigation (Mitchell, 2004).

Despite clear genetic control of nectar traits in mānuka, the levels of variation in nectar properties associated with floral stage and environment were at least as large as that contributed by genotype. A high level of variation in nectar chemistry, between flowers and plants, has been observed previously for other taxa, and is one of the most challenging aspects of nectar biology. Future measurements of mānuka nectar properties should control for developmental variation by careful selection of flowers of a consistent age or floral stage. The floral longevities reported here are long compared to previously reported values for wild mānuka (Primack, 1980), possibly because of the absence of flower visitors, the protected growth environment and the use of ornamental cultivars. Further research is needed to better understand how nectar composition varies with the age of the flower, position within the plant and timing within the flowering period of the plant. It is likely that nectar flow at the flower level varies predictably during the flowering period (Pleasants, 1983), and the timing and duration of flowering in mānuka is affected by genotype and the environment (Primack, 1980; Primack and Lloyd, 1980; Zieslin and Gottesman, 1986). In this study, nectar sugars per flower declined, and there was a change in ranking of the four high nectar-producing genotypes between the consecutive phenology and water stress experiments, even though the same plants were being sampled (compare sugars per flower and DHA/sugar between Fig. 4 and Table 2). Such genotype by environment interactions further contribute to variation in nectar traits, and reduce the heritability of traits subjected to artificial selection (Boose, 1997; Leiss and Klinkhamer, 2005).

The flow and composition of mānuka nectar was insensitive to plant water status, but was responsive to other environmental variables. Water stress has variable effects on nectar flow in other species, but typically reduces nectar volume without affecting sugar concentration (Villarreal and Freeman, 1990; Carroll *et al.*, 2001), and can affect the ranking of genotypes according to nectar volume and therefore sugar production (Leiss and Klinkhamer, 2005). While nectar volume was not measured directly in this study, water stress may reduce nectar volume in mānuka, rather than nectar sugar per flower, resulting in a more concentrated nectar. Nectar volume is difficult to measure reliably in mānuka, because of low volumes per flower and the open convex morphology of the nectary. The lack of a response in nectar composition to water stress does not support the hypothesis that the presence of DHA is linked to stress or osmoregulation. Insensitivity of total nectar sugars to water stress suggests that floral nectar sugar production is relatively independent of shoot water status and current leaf photosynthetic rates in mānuka, possibly reflecting the high stress tolerance and broad environmental range of the species (Stephens *et al.*, 2005; Savage *et al.*, 2016). A more severe or prolonged drought may be required before nectar flow declines (Burquez and Corbet, 1998). As frequently observed for herbaceous species (Pacini and Nepi, 2007), nectar flow in mānuka was positively correlated with temperature. Temporal lags between environmental conditions (temperature, radiation and humidity) and total nectar sugar or nectar volume have also been detected previously in other species (Shuel, 1952; Burquez and Corbet, 1998). The current experiment was not designed to test for environmental effects other than plant water status, making it difficult to distinguish between the effects of temperature, radiation and humidity. That an environmental response was detected in an experiment when glasshouse controls were operating to suppress day-to-day variation indicates that nectar flow in mānuka under natural conditions may be strongly influenced by temperature and other climatic variables associated with temperature.

Consistent changes in nectar flow and composition with floral stage of development provides insight into the mechanism of nectar production by the mānuka floral nectary. Sugar and DHA flows peaked soon after opening, between Stage 2 and 3, before declining as the flower ages, a pattern similar to other species characterized by a surge in nectar flow at anthesis. In floral nectaries of tobacco, Brassicaceae and other taxa the initial rise in nectar flow at anthesis is associated with the hydrolysis of starch stored in nectary plastids (Ren *et al.*, 2007; Ruhlmann *et al.*, 2010). Green plastids are abundant in the nectary tissue of mānuka flowers, but the role of plastids and starch in mānuka nectar secretion has not been investigated. After the initial rise, continuing nectar flow may be supported by sugar influx via the phloem (Ren *et al.*, 2007), and sugars derived from photosynthesis by the nectary itself may also contribute (Luttge, 2013). The DHA content of mānuka floral nectar peaked more sharply than the hexoses at Stage 3, and across all stages and genotypes, DHA to hexose ratios were more variable than the ratio of fructose to glucose, indicating that DHA production is not stochiometrically linked to synthesis of the larger sugars. It is possible that variation in the DHA content of mānuka nectar is connected to variation in the origin of nectary sugars over the functional lifespan of the nectary.

The decline in nectar sugars and DHA at Stage 4 also provides clear evidence for nectar reabsorption in mānuka. Nectar reabsorption by floral nectaries has been reported for other species, including *Eucalyptus*, another member of the Myrtaceae (Davis, 1997). Initially viewed as a resource recovery mechanism (Burquez and Corbet, 1991), reabsorption frequently occurs concurrently with nectar secretion and may therefore contribute to nectar homeostasis (Nepi and Stpiczynska, 2008). In three *Eucalyptus* species nectar reabsorption was non-selective for the three major sugars (glucose, fructose and sucrose), resulting in no change in nectar composition during reabsorption (Davis, 1997). In contrast, when reabsorption occurred in mānuka it appeared to be partially selective, with a gradual but significant increase in fructose to glucose ratio in older flowers of high nectar-producing genotypes. Like the hexoses, DHA is not volatile as a solid or from an aqueous solution (Epstein *et al.*, 2013). However, DHA disappeared faster from the standing nectar than the hexoses between Stages 3 and 4, either through reabsorption or consumption by an unknown process. The level of reabsorption of the hexoses in mānuka varied between genotypes, from no net reabsorption in WK to 50 % net reabsorption in RE. Individual flowers were sampled only once in this study, at the same time of day. Future research should also consider whether there are diurnal or circadian variations in nectar secretion and reabsorption rates, and whether nectar withdrawal stimulates increased nectar flow in mānuka, as observed in other species (Pacini and Nepi, 2007). Overall, it can be concluded that differences in the timing and rate of both secretion and reabsorption of individual nectar components contribute to both genotypic and temporal variation in nectar composition of mānuka. Selective secretion, reabsorption or loss of DHA compared to the hexoses further reinforces the conclusion that the triose sugar is produced and secreted separately from the hexoses.

The mechanisms of floral nectar sugar and DHA secretion in mānuka are unknown. The presence of sucrose in trace amounts supports a model of nectar sugars exiting secretory cells as sucrose via the efflux sucrose transporter SWEET9, followed by near complete hydrolysis to glucose and fructose by a cell wall invertase (Lin *et al.*, 2014). In the absence of hexose efflux carriers, it was hypothesized that nectar fructose/glucose ratios other than 1: 1 may be achieved by differential reabsorption of hexoses by active monosaccharide transporters that are known to be expressed in nectary tissue (Lin *et al.*, 2014). Genotype differences in sucrose content can arise from variable levels of invertase activity. Secretion of sucrose followed by hydrolysis may also explain why the ratio of fructose to glucose decreased with increasing residual levels of sucrose (Fig. 8). A higher rate of reabsorption of glucose would explain why the fructose to glucose ratio is higher than unity in mānuka nectar, and why the ratio increases when nectar is left to accumulate as flowers age.

In contrast to the larger sugars, DHA is not a commonly identified nectar component in other species, and its pathways for production, secretion and reabsorption in nectar are unknown. The lack of any relationship between nectar DHA and the relative contributions of the other sugars leads to the hypothesis that it originates from within the nectary parenchyma and enters the nectar independently of the SWEET9 sucrose efflux pathway. Alternative but less parsimonious explanations include synthesis by unidentified enzymes or microbial activity within the nectar itself. The most direct potential source from within secretory cells is as DHA phosphate or its isomer, glyceraldehyde 3-phosphate (together referred to as ‘triose phosphates’), central intermediates in glycolysis, gluconeogenesis and the pentose phosphate pathway. Glyceraldehyde 3-phosphate and DHA phosphate are rapidly and reversibly interconverted by the enzyme triose phosphate isomerase (TPI), and are the key product exported by chloroplasts during photosynthesis. DHA phosphate is therefore a precursor to cytosolic production of hexose phosphates and sucrose in photosynthetic cells in the light (MacRae and Lunn, 2006), and mānuka nectaries are green and photosynthetically active (M. Clearwater and S. Noe, University of Waikato, unpubl. res.). Photosynthesis may be contributing to nectar secretion in mānuka, as proposed for other species with green nectaries (Luttge, 2013). Similarly, Wenzler *et al.* (2008) concluded that pre-secretory nectar metabolism in *Anigozanthos* flowers involves partial cycling of sugars between the glycolytic and pentose phosphate pathways, via TPI and DHA phosphate. However, why an intermediate from these pathways might appear in its dephosphorylated form as a nectar component of mānuka is yet to be explained.

## SUPPLEMENTARY DATA

Supplementary data are available online at https://academic.oup.com/aob and consists of the following. Figs. S1–5. Effect of drying soil on predawn xylem water potential (Ψ_x dawn_) and nectar yield and composition for Stage 3 flowers of mānuka genotypes NT, RD, RE, SF and WK, respectively.

Supplementary Data
