# Supplementary material for: Influence of genotype, floral stage, and water stress on floral nectar yield and composition of mānuka (Leptospermum scoparium)
Source: Ann Bot. 2018 Jan 2;121(3):501–12. doi: 10.1093/aob/mcx183 (PMC5838834; doi:10.1093/aob/mcx183)
Supplement: Supplementary Data [file mcx183_suppl_supplementary_data.docx]

Supplementary Figure 1. Effect of drying soil on predawn xylem water potential (*Ψ*_x dawn_) and nectar yield and composition for stage 3 flowers of mānuka genotype NT. Irrigation was reduced in stressed plants (open symbols) from day 0, while non-stressed plants (closed symbols) were irrigated to soil field capacity daily. Stressed plants were returned to full irrigation on day 18. Values are means ±SE.

Supplementary Figure 2. Effect of drying soil on predawn xylem water potential (*Ψ*_x dawn_) and nectar yield and composition for stage 3 flowers of mānuka genotype RD. Irrigation was reduced in stressed plants (open symbols) from day 0, while non-stressed plants (closed symbols) were irrigated to soil field capacity daily. Stressed plants were returned to full irrigation on day 18. Flower numbers for this genotype were too low to allow continued nectar collection after day 13. Values are means ±SE.

Supplementary Figure 3. Effect of drying soil on predawn xylem water potential (*Ψ*_x dawn_) and nectar yield and composition for stage 3 flowers of mānuka genotype RE. Irrigation was reduced in stressed plants (open symbols) from day 0, while non-stressed plants (closed symbols) were irrigated to soil field capacity daily. Stressed plants were returned to full irrigation on day 18. Values are means ±SE.

Supplementary Figure 4. Effect of drying soil on predawn xylem water potential (*Ψ*_x dawn_) and nectar yield and composition for stage 3 flowers of mānuka genotype SF. Irrigation was reduced in stressed plants (open symbols) from day 0, while non-stressed plants (closed symbols) were irrigated to soil field capacity daily. Stressed plants were returned to full irrigation on day 18. Levels of nectar sugars for this genotype were frequently close to or below the levels of detection. Values are means ±SE.

Supplementary Figure 5. Effect of drying soil on predawn xylem water potential (*Ψ*_x dawn_) and nectar yield and composition for stage 3 flowers of mānuka genotype WK. Irrigation was reduced in stressed plants (open symbols) from day 0, while non-stressed plants (closed symbols) were irrigated to soil field capacity daily. Stressed plants were returned to full irrigation on day 18. Note the difference in vertical scale for E (sucrose) between this and the Figures for the other genotypes. Values are means ±SE.
